# Identification of miRNAs and Their Target Genes Involved in Cucumber Fruit Expansion Using Small RNA and Degradome Sequencing

**DOI:** 10.3390/biom9090483

**Published:** 2019-09-12

**Authors:** Yongdong Sun, Weirong Luo, Huaicheng Chang, Zhenxia Li, Junguo Zhou, Xinzheng Li, Jinliang Zheng, Mingxian Hao

**Affiliations:** 1 School of Horticulture and Landscape Architecture, Henan Institute of Science and Technology, Xinxiang 453003, China; rowe0803@163.com (W.L.); 15617199956@163.com (H.C.); lizhenxia196@163.com (Z.L.); junguo1020@163.com (J.Z.); lxz2283@126.com (X.L.); jin100liang@126.com (J.Z.); haomingxian6975555@163.com (M.H.); 2 Henan Province Engineering Research Center of Horticultural Plant Resource Utilization and Germplasm Enhancement, Xinxiang 453003, China

**Keywords:** cucumber (*Cucumis sativus* L.), fruit expansion, miRNA, sRNA and degradome sequencing, qRT-PCR

## Abstract

Fruit expansion is an essential and very complex biological process. Regulatory roles of microRNAs (miRNAs) and miRNA–mRNA modules in the cucumber fruit expansion are not yet to be investigated. In this work, 1253 known and 1269 novel miRNAs were identified from nine cucumber fruit small RNA (sRNA) libraries through high-throughput sequencing. A total of 105 highly differentially expressed miRNAs were recognized in the fruit on five days post anthesis with pollination (EXP_5d) sRNA library. Further, expression patterns of 11 differentially expressed miRNAs were validated by quantitative real-time PCR (qRT-PCR). The expression patterns were similar to sRNAs sequencing data. Transcripts of 1155 sequences were predicted as target genes of differentially expressed miRNAs by degradome sequencing. Gene Ontology (GO) enrichment showed that these target genes were involved in 24 biological processes, 15 cell components and nine molecular functions. Kyoto Encyclopedia of Genes and Genomes (KEGG) pathway analysis demonstrated that these target genes were significantly enriched in 19 pathways and the enriched KEGG pathways were associated with environmental adaptation, signal transduction and translation. Based on the functional prediction of miRNAs and target genes, our findings suggest that miRNAs have a potential regulatory role in cucumber fruit expansion by targeting their target genes, which provide important data for understanding the miRNA-mediated regulatory networks controlling fruit expansion in cucumber. Specific miRNAs could be selected for further functional research and molecular breeding in cucumber.

## 1. Introduction

Cucumber (*Cucumis sativus* L.) is one of the most common vegetable crops in China as well as worldwide. Globally, 84 million tons of cucumber fruit were produced from 2.27 million ha cultivated area. China has the highest cultivated area and is the top producer of cucumber in the world [1]. Fruit expansion in cucumber is often suppressed by environmental factors such as low temperature, weak light and the absence of male flowers in the case of greenhouses of North China. While in South China, the rains during pollination and fruit expansion severely affect the fruit expansion and thereby lower the production and quality of cucumber fruit.

Fruit expansion is an essential biological phase and involves several complex physiological and molecular processes. In the present day, the fruit expansion process has been well explored at the phytohormone level and it has been reported that phytohormones such as auxins, cytokinins and gibberellic acid (GA) have crucial roles in fruit set and expansion. In nature, the non-parthenocarpic fruit expansion is induced by pollination, which in turn increases the phytohormone level in the ovary [2,3,4]. Boonkorkaew et al. reported that pollination stimulated the synthesis of cytokinins and auxins in the cucumber fruit leading to cell division and fruit development [5]. The fruit expansion could also be induced artificially by the application of exogenous phytohormones. Previous studies have reported that auxins, cytokinins, GA and brassinosteroids (BRs) are widely used to induce parthenocarpic fruit by promoting fruit set and expansion in cucumber [3,6,7,8]. In recent years, developmental genetic studies have uncovered the fruit development at the gene expression level. A number of genes controlling fruit development such as auxin response factor (*ARF*) [9], expansin gene [10], D-type cyclin gene [11,12], auxin signaling F-box (*AFB*) [13], kinesin gene (*KF*) [14], cyclin dependent kinases [15], GDSL-motif lipase/hydrolase family protein gene [15] and lipid transfer protein family protein gene [15] were identified and analyzed. In spite of the accumulated information, the molecular regulatory mechanism of cucumber fruit expansion remains unknown.

MicroRNAs (miRNAs) in plants are endogenous, 20–24 nucleotide (nt) length, noncoding, small RNAs (sRNAs) that play various roles in regulating the expression of functional genes at post-transcriptional and translational levels either by targeting mRNAs [16,17] or inhibiting translation [18,19]. Plant miRNAs are involved in a series of biological processes, such as growth and development [20,21], hormone signal transduction [22,23] and response to abiotic and biotic stresses [24,25]. Furthermore, plant miRNAs also participate in a diverse part of fruit development and play fundamental regulatory roles, including fruit initiation, fruit size, fruit coloration, fruit ripening and other fruit developmental processes [26]. Distinctive miRNAs are expressed differentially at different fruit developmental stages [27]. miR156-targeted *SPL*/*SBP* box transcription factors regulate ovary and fruit development by controlling fruit development initiation [28] and miR390-*TAS*-*ARF* is essential in regulating the auxin signaling pathway involved in the tomato fruit developmental process [29]. Overexpression of miR172 induces small-sized parthenocarpic fruit by affecting fruit development and size formation in tomato [30]. miR393 is involved in the fruit set and development processes of cucumber by the post-transcriptional regulation of *CsTIR1* and *CsAFB2* [31]. Zhang et al. predicted that miR159, miR164, miR319, miR393 and miR396 may be involved in young fruit development of Hami melon (*Cucumis melo*) by investigating the miRNAs expression profiles during the fruit development process [32]. Ye et al. analyzed the miRNAs expression profiles between cucumber fruit and a mixture of leaves, stems, and roots by means of high-throughput sequencing, and recognized 166 miRNAs in cucumber [33]. Apparently, there are few reports on miRNAs regulation in cucumber fruit expansion and the miRNA–mRNA modules in cucumber expanding fruit are still not well understood.

To understand the regulatory roles of miRNAs and underlying molecular regulatory networks in cucumber fruit expansion, the expression profiles of miRNAs at three developmental stages of cucumber fruit were studied through high-throughput sequencing in this work. Expression levels of the differentially expressed miRNAs were validated by quantitative real-time PCR (qRT-PCR) analysis. Additionally, the target genes of miRNAs were identified from three developmental stages of cucumber fruit via degradome sequencing. In this study, miRNAs and their target genes related to cucumber fruit expansion were obtained. The regulatory roles of miRNAs and their target genes were predicted by Gene Ontology (GO) and the Kyoto Encyclopedia of Genes and Genomes (KEGG). The findings provided important data for understanding the regulatory roles of miRNAs and clarifying the miRNA-mediated regulatory networks controlling fruit expansion in cucumber.

## 2. Materials and Methods

### 2.1. Plant Materials

Cucumber inbred line *Cs*0401 was used for this work. The experiment was carried out in the Vegetable Science Research Laboratory and the plastic greenhouse, Henan Institute of Science and Technology, Xinxiang, China. Cucumber seeds of uniform size were selected and soaked in 55 °C water for 15 min. Then the seeds were cultured in glass dishes at 28 ± 1 °C to induce germination in the Vegetable Science Research Laboratory. Germinating seeds were sown in plug trays filled with a 2:1 (*v*/*v*) mixture of peat and vermiculite. When the second true leaf had developed completely, cucumber seedlings were transferred to the plastic greenhouse. Only one female flower was kept in one plant to maintain the growth consistency. The male and female flowers were clipped to prevent natural pollination two days before anthesis. In the pollination treatment, female flowers were hand-pollinated on the day of anthesis. Ovaries or fruits were collected from each plant at three stages: ovary on the day of anthesis without pollination (uE_0 d), ovary on five days post anthesis without pollination (uE_5 d) and fruit on five days post anthesis with pollination (EXP_5 d), respectively. Each treatment was replicated three times and included ten ovaries or fruits per replication. All the collected ovaries or fruits were immediately frozen in liquid nitrogen, then stored at −80 °C for RNA isolation.

### 2.2. sRNA Library Construction and Sequencing

Total RNA from uE_0 d, uE_5 d and EXP_5 d was isolated separately using Trizol reagent (Invitrogen, Shanghai, China) following the manufacturer’s instructions. Genomic DNA contamination of RNA samples was removed using RNase-free DNase Kit (Promega, Madison, WI, USA). The quantity and purity of total RNA were analyzed by Bioanalyzer 2100 (Agilent, Palo Alto, CA, USA). Approximately 1 μg total RNA without genomic DNA contamination was used for the sRNA library construction, according to the protocol of TruSeq Small RNA Sample Prep Kits (Illumina, San Diego, CA, USA). As a result, nine cucumber fruit sRNA libraries were constructed. The libraries were sequenced on an Illumina Hiseq2500 at the LC-Bio Co., Ltd. (Hangzhou, China).

### 2.3. Bioinformatics Analysis of Sequencing Data

Low quality reads and the adapter contaminants were discarded from the raw data after the high-throughput sequencing. Then the clean reads with 18–25 nt RNAs were chosen for a blast search against Rfam (http://www.sanger.ac.uk/Software/Rfam) and GenBank (http://www.ncbi.nlm.nih.gov/GenBank/) databases to eliminate rRNAs, tRNAs, small nucleolar RNAs (snoRNAs) and small nuclear RNAs (snRNAs). The remaining data were aligned with all the known miRNA sequences in plants from miRBase 22.1 (http://www.mirbase.org) to identify conserved miRNAs produced by cucumber fruit. The unannotated sRNA sequences were aligned to the cucumber genome database (http://cucumber.genomics.org.cn/) to obtain miRNA precursors. The miRNA that showed similarity (no more than three mismatches) to the known miRNA sequences in plants was classified as conserved miRNA, the remaining ones were classified as novel miRNAs.

### 2.4. Differential Expression Analysis of miRNAs

To compare the differentially expressed miRNAs at three fruit developmental stages, expression levels of miRNAs were normalized by transcripts per million (TPM). Furthermore, the differentially expressed miRNAs were analyzed according to the formula: Normalized expression (NE) = Actual miRNA reads count / Total count of clean reads × 1,000,000. The miRNAs with a fold change in the NE greater than 1.5 or less than −1.5, along with *p*-value *≤* 0.05 were considered as differentially expressed ones during cucumber fruit expansion.

### 2.5. qRT-PCR Analysis

Expression levels of the differentially expressed miRNAs were validated by qRT-PCR analysis. Eleven miRNAs (csa-miR156a, csa-miR160a, cme-miR164a, csa-MIR166d, csa-MIR319b, csa-MIR390b, csa-MIR395b, csa-miR395c, csa-MIR399a, cme-miR399c and csa-mir013) were selected for qRT-PCR validation. Reverse transcription was performed with PrimeScript™ RT Reagent Kit (Takara, Dalian, China). qRT-PCR analysis was performed with the ABI Step One Plus PCR System and SYBR Premix Ex Taq^TM^ II (TaKaRa, Dalian, China) according to the manufacturer’s instructions, using *U6* as the reference gene for miRNAs. Triplicate quantitative assays were carried out for each sample with a gene-specific primer (Table 1).

### 2.6. Degradome Sequencing

To obtain target genes of the differentially expressed miRNAs, three cucumber fruit degradome cDNA libraries from the uE_0 d, uE_5 d and EXP_5 d were constructed and sequenced on Illumina Hiseq 2500 at the LC-Bio Co., Ltd. (Hangzhou, China). Clean reads of the degradome sequencing were obtained by removing low quality reads and the adapter contaminants. Then the clean reads of the degradome data were mapped to the cucumber genome database. CleaveLand pipeline 4.3 (https://sites.psu.edu/axtell/software/cleaveland4/) was used to confirm the potential miRNA:target pairs with default parameters. To predict the function of the miRNA and their targets, GO enrichment (http://www.Geneontology.org/) and KEGG pathway (http://www.kegg.jp/kegg/) were carried out for all the annotated miRNAs and their target genes, respectively.

## 3. Results

### 3.1. Overview of miRNAs Sequencing

**Figure 1:**
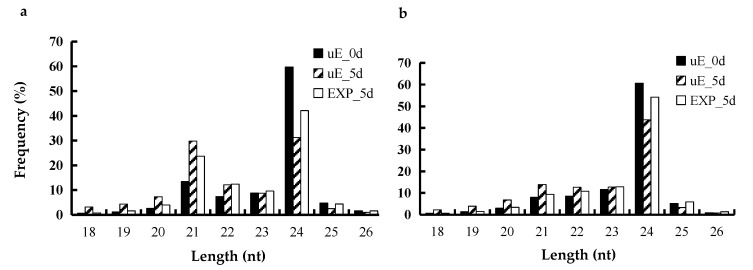
Length distribution of sRNA sequences in three cucumber fruit libraries. Note: (**a**) total sRNA; (**b**) unique sRNA.

To investigate the regulatory role of miRNAs during cucumber fruit expansion, nine cucumber fruit sRNA libraries were constructed from three stages: uE_0 d, uE_5 d and EXP_5 d, respectively. A total of 19,718,088 (uE_0d), 20,035,416 (uE_5d) and 22,463,142 (EXP_5d) raw sRNA reads were generated through high-throughput sequencing, and 16,698,831 (uE_0d), 15,521,125 (uE_5d) and 18,989,300 (EXP_5d) valid reads were acquired after removing low quality reads and the adapter contaminants, respectively (Table 2). The 18–25 nt RNAs were annotated and 4,366,701 (uE_0d), 3,472,867 (uE_5d) and 3,749,311 (EXP_5d) valid unique sRNA reads were identified from the sRNA libraries (Table 2). The length distribution of the sRNA sequences was then analyzed (Figure 1). More than 80% of the total and unique sRNA sequences were between 21 to 24 nt in length, which was the typical size range generated by Dicer. The 24 nt total sRNA was the most abundant in uE_0d, uE_5d and EXP_5d sRNA libraries, accounting for 59.73%, 31.19% and 42.08% of the total reads, respectively (Figure 1a). Among the unique sequences, 24 nt unique sRNA represented the greatest proportion of sequences in the three sRNA libraries, accounting for 60.62%, 43.80% and 54.15%, respectively (Figure 1b). Based on the results, 24 nt sRNA was the most abundant sRNA in cucumber fruit.

### 3.2. Identification of Known and Novel miRNAs

In order to identify known and novel miRNAs during cucumber fruit expansion, the unique clean reads recovered from the cucumber fruit sRNA libraries were compared with the known miRNAs in miRBase 22.1. As a consequence, 2522 miRNAs including 1253 known and 1269 novel ones were revealed from the cucumber fruit sRNA libraries in this study (Table S1). These conserved miRNAs were aligned with *Cucumis melo*, soybean, apple, grape and other plant species. In addition, miRNA sequencing conducted in the present study confirmed that a total of 92 miRNAs including 85 known and seven novel miRNAs were highly expressed in the cucumber fruit sRNA libraries, and 47 known miRNAs sequences recognized here were primarily aligned with *Cucumis melo*, which may be due to the strong homology between cucumber and *Cucumis melo* (Table S1).

### 3.3. Differential Expression of miRNAs

**Figure 2:**
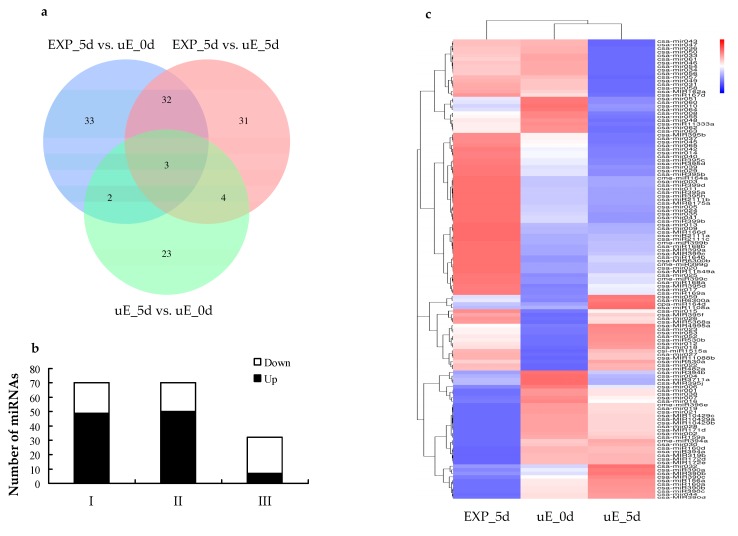
Differentially expressed miRNAs at three developmental stages. (**a**) distribution of differentially expressed miRNAs; (**b**) numbers of differentially expressed miRNAs. I: EXP_5d vs. uE_0d; II: EXP_5d vs. uE_5d; III: uE_5d vs. uE_0d; white and black indicates the numbers of down-regulated and up-regulated miRNAs, respectively; (**c**) heatmap of differentially expressed miRNAs. Red and blue indicates the expression level. The original expression values of miRNAs are normalized using Z-score normalization.

To recognize the miRNAs involved in cucumber fruit expansion, miRNAs expression abundance with fold change in the NE greater than 1.5 or less than −1.5 and *p*-value ≤ 0.05 were as thresholds for differentially expressed miRNAs. Expression difference of miRNAs between EXP_5d vs. uE_0d, EXP_5d vs. uE_5d and uE_5d vs. uE_0d were analyzed using DESeq to compare the log_2_ transformed read counts. Of the miRNAs, 70, 70 and 32 exhibited remarkable expression difference between EXP_5d and uE_0d, between EXP_5d and uE_5d, and between uE_5d and uE_0d, respectively (Figure 2a,c, Table S2). Forty-nine of the 70 differentially expressed miRNAs were responsible for up-regulated and 21 were for down-regulated in EXP_5d compared to uE_0d (Figure 2b). Out of the miRNAs between EXP_5d and uE_0d, the highest up-regulated miRNA was csa-mir005 (4.72-fold), while the most down-regulated miRNA was csa-MIR319b (4.55-fold) (Table S2a). Fifty miRNAs were up-regulated and 20 were down-regulated in EXP_5d compared to uE_5d (Figure 2b). Among the miRNAs between EXP_5d and uE_5d, the most up-regulated miRNA was csa-MIR395b (6.40-fold), while the most down-regulated miRNAs were csa-MIR319b (4.72-fold) and csa-miR160d (4.09-fold) (Table S2b). Seven miRNAs were up-regulated and 25 were down-regulated in uE_5d compared to uE_0d (Figure 2b). In case of the miRNAs between uE_5d and uE_0d, the most up-regulated miRNA was cpa-miR164d (3.08-fold), while the most down-regulated miRNAs were csa-mir010 (4.40-fold) and csa-miR008 (4.28-fold) (Table S2c). At last, a total of 128 highly differentially expressed miRNAs were recognized between EXP_5d vs. uE_0d, EXP_5d vs. uE_5d and uE_5d vs. uE_0d sRNA libraries. There were 105 highly differentially expressed miRNA recognized between EXP_5d vs. uE_0d and EXP_5d vs. uE_5d sRNA libraries and among them, 58 miRNAs were known, and 47 miRNAs were novel. The results suggested that 105 highly differentially expressed miRNAs in EXP_5d sRNA library have important regulatory roles in cucumber fruit expansion.

### 3.4. Validation of miRNA Expression by qRT-PCR

**Figure 3:**
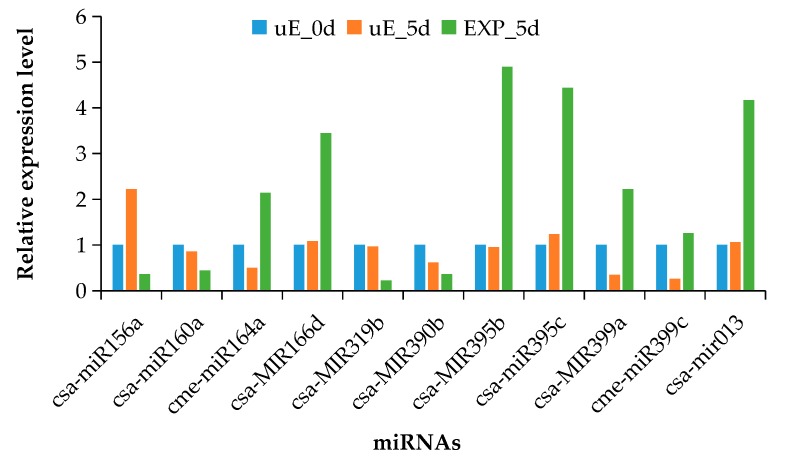
Expression validation of 11 differentially expressed miRNAs at three fruit developmental stages by qRT-PCR. Note: Expressional level of each miRNA in uE_0d is set as one and fold change of each miRNA relative to uE_0d is calculated.

To validate the sRNA sequencing results, we investigated the expression levels of the miRNAs at three fruit developmental stages using qRT-PCR. Based on the sRNA sequencing results, 10 known miRNAs (including six up-regulated and four down-regulated) and one novel miRNA (up-regulated), which was differentially expressed in the EXP_5d sRNA library, were used for qRT-PCR analysis. The results demonstrated that expression trends of 11 selected miRNAs obtained by qRT-PCR were similar with the sRNAs sequencing data (Figure 3), however, the exact fold changes varied between sRNA sequencing data and qRT-PCR, which may be due to differences of the two approaches in the sensitivity and specificity. The results illustrated that the sRNA sequencing data in this study obtained was reliable. On the other hand, the results also showed that these 11 differentially expressed miRNAs were related to cucumber fruit expansion.

### 3.5. Identification of Target Genes

To clarify the miRNA-mediated regulatory networks during cucumber fruit expansion, we identified the target genes of miRNAs by constructing three degradome libraries (uE_0d, uE_5d and EXP_5d). After degradome sequencing, 41.92, 32.01 and 36.96 million raw reads were obtained from the uE_0d, uE_5d and EXP_5d degradome libraries, respectively. After removing the adaptor reads, 6.62 (75.58%), 4.79 (67.91%) and 5.67 (76.29%) million unique reads were mapped to the cucumber genome data, which suggested that some of the filtered reads mapped to unannotated genes (Table S3). Further, a total of 1155 transcripts were predicted as target genes of identified differentially expressed miRNAs. Among them, the maximum target gene number for one miRNA was 224 and minimum target gene number for one miRNA was one. On the other hand, the maximum miRNA number for one transcript was five and minimum miRNA number for one transcript was one (Table S4). Degradome sequencing revealed that these 11 differentially expressed miRNAs had 102 target genes (Table S4). Target genes of some differentially expressed miRNAs involved in cucumber fruit expansion were in Table 3.

### 3.6. The Analysis of GO and KEGG

**Figure 4:**
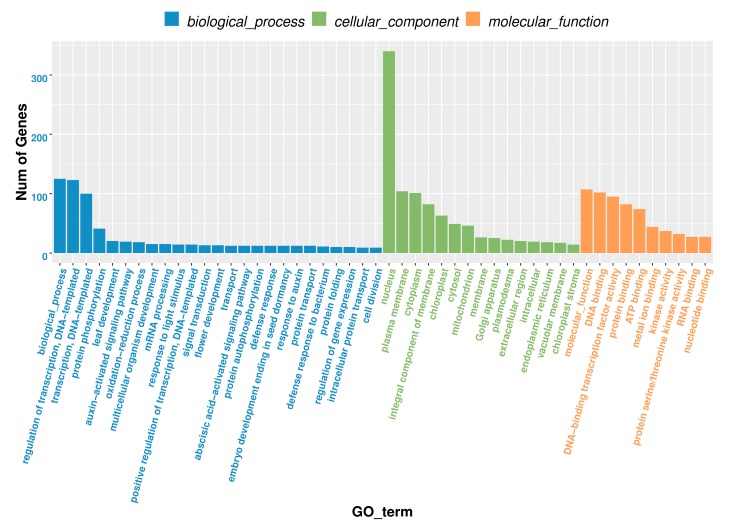
Gene Ontology (GO) analysis of target genes of differentially expressed miRNAs.

**Figure 5:**
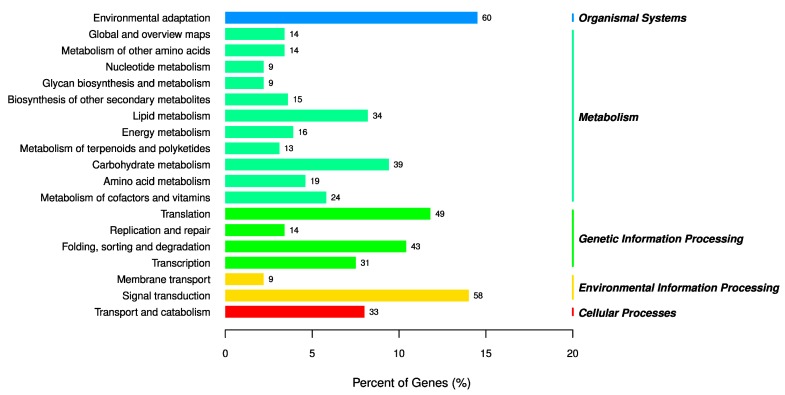
Kyoto Encyclopedia of Genes and Genomes (KEGG) analysis of target genes of differentially expressed miRNAs.

To better understand the functions of differentially expressed miRNAs in cucumber fruit expansion and illustrate the miRNA–mRNA modules, the target genes of differentially expressed miRNAs were functionally annotated with GO. The results indicated that the target genes of the differentially expressed miRNAs were enriched in 24 biological processes, 15 cell components and nine molecular functions (Figure 4). In biological processes, the predominant terms were related to regulation of transcription, DNA-templated (GO:0045892) and transcription, DNA-templated (GO:0006355). Nucleus (GO:0005634), plasma membrane (GO:0005886) and cytoplasm (GO:0005737) were found to be the most abundant in the cellular component category. DNA binding (GO:0003677) and DNA-binding transcription factor activity (GO:0003700) were the two most enriched molecular functions. In general, GO analysis indicates that the target genes identified here might be involved in various physiological processes during cucumber fruit expansion processes, especially regulation of transcription. Based on the KEGG pathway database, the identified target genes were significantly enriched in metabolism, genetic information processing, environmental information processing, organismal systems and cellular processes, involved in 19 pathways (Figure 5). The enriched KEGG pathways in cucumber fruit expansion were mostly found to be associated with environmental adaptation, signal transduction and translation.

## 4. Discussion

miRNAs participate in the cucumber fruit development [26], however, there is very limited information available about miRNAs and their target genes during the expansion process of cucumber fruit. Therefore, in the current study, miRNAs and their target genes related to cucumber fruit expansion were revealed in terms of sRNA sequencing and degradome sequencing.

sRNA sequencing provides incredible amounts of data and efficiently explores known and novel miRNAs. A total of 19,718,088 (uE_0d), 20,035,416 (uE_5d) and 22,463,142 (EXP_5d) raw sRNA sequences were identified in this study (Table 2). An analysis of the distribution of clean read lengths revealed that 24 nt was the most common sRNA length (Figure 1). The result was consistent with the previous reports in cucumber [33,34]. In the present study, a total of 92 miRNAs including 85 known and seven novel ones were highly expressed in the cucumber fruit sRNA libraries. Seventy miRNAs each were differentially expressed in EXP_5d vs. uE_0d as well as EXP_5d vs. uE_5d, respectively. Finally, a total of 105 highly differentially expressed miRNAs were recognized in the EXP_5d sRNA library. Our results demonstrated that these highly differentially expressed miRNAs in the EXP_5d sRNA library were associated with cucumber fruit expansion, which supported that miRNAs are involved in controlling the fruit development [26] and provided important data for understanding the regulatory roles and networks of miRNAs during the cucumber fruit expansion. miRNA-mediated regulatory roles would be uncovered by analyzing their spatial and temporal expression patterns [35]. Previous reports suggest that miRNAs have differential expression in specific developmental stages [36] and play important regulatory roles in controlling fruit development [26,31,32]. In this study, 105 highly differentially expressed miRNAs were recognized in EXP_5d sRNA library and expression levels of 11 miRNAs among them were detected at three fruit developmental stages through qRT-PCR. The results showed that the expression of 11 selected miRNAs were different at three developmental stages (Figure 3), which verified the reports of Sunkar et al. [36]. On the other hand, the expression trends of miRNAs obtained by qRT-PCR were similar to the results of the sRNA sequencing data in this work, which confirmed the reliability of sRNA sequencing data.

Cucumber fruit development is regulated by complex gene networks. Earlier, Bartel reported that miRNAs can mediate gene expression essentially through miRNA-guided cleavage of target genes in plants [37]. Therefore, the identification of miRNA target genes is crucial to reveal the regulatory roles of miRNAs. We have identified the target genes of miRNAs from the uE_0 d, uE_5 d and EXP_5 d degradome libraries. GO analysis showed that these identified target genes were enriched in 24 biological processes, 15 cell components and nine molecular functions (Figure 4). Regulation of transcription, DNA-templated (GO:0045892) and transcription, DNA-templated (GO:0006355) were the most predominant terms in biological processed. Ye et al. reported that most of miRNA target genes related to biological processes were transcription factors and functional proteins involved in plant metabolism and plant hormones [33]. Target genes of miRNAs are usually transcription factors in plants [38]. Our findings were consistent with previous reports in cucumber of Ye et al. [33].

miRNA-mediated regulatory networks participate in a diverse part of fruit development and play fundamental regulatory roles in plants. miR156 regulates the initiation of tomato fruit development by targeting *SPL*/*SBP*-box transcription factors [28]. In Hami melon fruit, cme-miR156a-e/g-j were up-regulated at fruit ripening stage compared to the early stage, which indicated that down-regulation of miR156 could promote developmental transition [32]. In the current investigation, csa-miR156a was down-regulated in expanding cucumber fruit compared to nonexpanding ovary (Figure 3), which showed that down-regulation of csa-miR156a was involved in cucumber fruit development and regulated the fruit expansion. Our research corresponded with the results of Silva et al. [28] and Zhang et al. [32]. miR160 is related to many plant biotic processes, such as flower identity specification, leaf development, fruit formation and other plant processes [17]. Down-regulation of miR160 inhibits the abscission of petal, anther and fruit in tomato, resulting in elongated and pear-shaped fruit compared to the control [39]. Ectopic expression of miR160-insensitive *SlARF10A* induces narrow leaflet blades, sepals and petals, and abnormally shaped fruit [40]. csa-miR160a was down-regulated in expanding cucumber fruit compared to nonexpanding ovary (Figure 3) and the target gene of csa-miR160a was auxin response factor 7 (Table 3). A similar target gene of miR160 was found by Damodharan et al. [39] and Hendelman et al. [40]. The results indicated the possible roles of csa-miR160a targeting auxin response factor 7 in controlling cucumber fruit expansion. miR164 was involved in tissue differentiation during plant development, together with its target genes *NAC* domain transcription factors [41]. miR164 that showed higher expression during fruit development in prickly pear cactus probably performs different functions by regulating different *NAC* transcription factors [42]. However, its expression started at a low level and then increased during tomato fruit development [27]. In this study, cme-miR164a exhibited a higher expression level in expanding cucumber fruit consisting of nonexpanding ovary (Figure 3), and its target genes were 1-phosphatidylinositol-3-phosphate 5-kinase FAB1B-like and NAC domain-containing protein 89-like (Table 3), which suggested that cme-miR164a had a new function in cucumber fruit expansion by regulating its target genes. miR166 participates in axillary meristem initiation and leaf development by targeting HD-Zip transcription factors *PHB* and *PHV* [43,44]. In addition, higher expression levels of miR166 at fruit ripening stage in both tomato [45] and banana [46] suggest that it is a potentially important regulator of fruit ripening. In the current study, compared to nonexpanding ovary, csa-MIR166d had a higher expression level in expanding cucumber fruit (Figure 3), and its target gene was *ABC* transporter *C* family member 15 (Table 3); there was no sign of this miRNA targeting *PHB* or *PHV.* Perhaps it is because csa-MIR166d sequence is not the functionally conserved and mature one. miR319 is one of the most abundant miRNAs and plays an important role at an early developmental stage in tomato fruit [27]. Zhang et al. [32] reported that cme-miR319 together with its target genes *MYB* and *TCP* transcription factor may play an important role during the young fruit development in Hami melon. csa-MIR319b was highly down-regulated in expanding cucumber fruit, compared to nonexpanding ovary (Figure 3), which suggested that csa-MIR319b regulated the cucumber fruit expansion. miR390 targets *TAS3* gene. *TAS3*-derived siRNAs target the *ARF2*, *ARF3*, and *ARF4* [47,48]. The miR390-*TAS*3-*ARF* pathway is mainly related to the regulation of leaf and flower development, especially the leaf morphogenesis in *Arabidopsis thaliana*, tomato and tobacco [49,50]. csa-MIR390b was down-regulated in expanding cucumber fruit, compared to nonexpanding ovary (Figure 3), which demonstrated the down-regulation of csa-MIR390b promoted cucumber fruit expansion. miR399 targets E2 ubiquitin conjugase related protein that negatively affectes phosphate (Pi) content and remobilization [51]. In woodland strawberry fruit, overexpressing miR399a can significantly improve fruit quality by increasing the soluble solids and regulation of Pi homeostasis [52]. In this work, the expression levels of csa-MIR399a and cme-miR399c were higher in expanding cucumber fruit than in nonexpanding ovary (Figure 3), and the target gene of cme-miR399c was ubiquitin-conjugating enzyme E2 24 (Table 3), which was in agreement with the report of Bari et al. [51]. However, there was no report about the function of miR399 in fruit expansion in the previous research. Our findings suggested that miR399 potentially participated in the cucumber fruit expansion. Additionally, the novel miRNA (csa-mir013) exhibited a higher expression level in expanding cucumber fruit consisting of nonexpanding ovary (Figure 3), and 40 target genes were detected, including signal recognition particle subunit SRP72-like, cell division control-like protein, mitogen-activated protein kinase homolog D5-like and so on (Table S4). We speculate that csa-mir013 and their target genes likely act in a species-specific manner during cucumber fruit expansion. Overall, these findings will facilitate the understanding of the regulatory roles of miRNAs and miRNA-mediated regulatory networks during cucumber fruit expansion.

## 5. Conclusions

In summary, 1253 known and 1269 novel miRNAs were identified in the nine sRNA libraries through sRNA sequencing. There were 70 differentially expressed miRNAs (|log_2_ (fold change) | ≥ 1.5 and *p*-value ≤ 0.05) both in EXP_5d vs. uE_0d and EXP_5d vs. uE_5d sRNA libraries, whilst only 32 miRNAs in uE_5d vs. uE_0d. Finally, a total of 105 highly differentially expressed miRNAs were recognized in EXP_5d sRNA library. Expression patterns of 11 miRNAs highly differentially expressed in the EXP_5d sRNA library were validated by qRT-PCR. The results were similar to the sRNA sequencing data. A total of 1155 transcripts were identified as target genes of miRNAs. GO analysis showed that the target genes identified here might be involved in various physiological processes during cucumber fruit expansion processes, especially regulation of transcription. KEGG pathway analysis showed that these target genes were significantly enriched in 19 pathways and the enriched KEGG pathways were associated with environmental adaptation, signal transduction and translation. Based on the functional prediction of miRNAs and their target genes, it suggested that miRNAs and their target genes had a potential regulatory role in cucumber fruit expansion. Specific miRNAs could be selected for further functional research and molecular breeding in cucumber.

## Figures and Tables

**Table 1:** Primers sequences for 11 selected microRNAs (miRNAs).

Primer Name	Primer Sequence (5’–3’)
*U6*-R	GGGGACATCCGATAAAATTGG
*U6*-F	GATTTGTGCGTGTCATCCTT
csa-miR156a-RT	GTCGTATCCAGTGCAGGGTCCGAGGTATTCGCACTGGATACGACCAATGT
csa-miR156a-F	CCGGCGTGACAGAAGAGAGT
csa-miR160a-RT	GTCGTATCCAGTGCAGGGTCCGAGGTATTCGCACTGGATACGACAGGCAT
csa-miR160a-F	CGTGCCTGGCTCCCTGT
cme-miR164a-RT	GTCGTATCCAGTGCAGGGTCCGAGGTATTCGCACTGGATACGACAGCACG
cme-miR164a-F	ACGGTGGAGAAGCAGGGC
csa-MIR166d-RT	GTCGTATCCAGTGCAGGGTCCGAGGTATTCGCACTGGATACGACATCTCG
csa-MIR166d-F	GCCGAATGTTGTCTGGTGC
csa-MIR319b-RT	GTCGTATCCAGTGCAGGGTCCGAGGTATTCGCACTGGATACGACTGAGCC
csa-MIR319b-F	TCGCAGCTGCTGACTCGTT
csa-MIR390b-RT	GTCGTATCCAGTGCAGGGTCCGAGGTATTCGCACTGGATACGACAAAACT
csa-MIR390b-F	TTCCGGCGCTATCTATCCTG
csa-MIR395b-RT	GTCGTATCCAGTGCAGGGTCCGAGGTATTCGCACTGGATACGACGATGAA
csa-MIR395b-F	TGGCGGAGTTTCCCTGAAT
csa-miR395c-RT	GTCGTATCCAGTGCAGGGTCCGAGGTATTCGCACTGGATACGACAGAGTT
csa-miR395c-F	GCGGCTGAAGTGTTTGGG
csa-MIR399a-RT	GTCGTATCCAGTGCAGGGTCCGAGGTATTCGCACTGGATACGACTCTGCC
csa-MIR399a-F	GTGGCGGGGCAATTACTCT
cme-miR399c-RT	GTCGTATCCAGTGCAGGGTCCGAGGTATTCGCACTGGATACGACCCGGGC
cme-miR399c-F	AGCGGTGCCAAAGGAGATT
csa-mir013-RT	GTCGTATCCAGTGCAGGGTCCGAGGTATTCGCACTGGATACGACAAAAAA
csa-mir013-F	GCGGTGGAGGGTCGAATT

**Table 2:** Statistics of reads from cucumber fruit small RNA (sRNA) libraries.

Sequence Type	uE_0d		uE_5d		EXP_5d	
	Total sRNA Number	Unique sRNA Number	Total sRNA Number	Unique sRNA Number	Total sRNA Number	Unique sRNA Number
Raw reads	19,718,088	5,363,469	20,035,416	4,862,572	22,463,142	4,840,446
3ADT and length filter	1,026,683	692,365	1,664,644	1,001,119	756,053	751,373
Junk reads	65,726	32,144	53,301	24,553	104,331	42,517
Rfam	1,295,187	84,578	2,077,132	92,228	1,922,733	106,090
mRNA	633,103	188,082	738,616	273,020	695,312	191,756
Repeats	4849	1546	5029	2307	3974	1505
rRNA	1,038,125	55,560	1,746,084	61,791	1,607,783	71,409
tRNA	121,383	17,572	90,316	14,269	142,211	21,113
snoRNA	28,947	2787	38,706	4733	46,098	3276
snRNA	35,054	3965	91,704	5469	60,823	4781
other Rfam RNA	71,679	4694	110,322	5967	65,818	5511
valid reads	16,698,831	4,366,701	15,521,125	3,472,867	18,989,300	3,749,311

**Table 3:** Target genes of some differentially expressed miRNAs involved in cucumber fruit expansion.

Small RNA	Transcript	Transcript Annotation	log_2_ (Fold Change)	Degradome Cleavage Site
csa-miR160a	XM_011657993.1	auxin response factor 7-like	−1.30	1681
csa-miR160a	XM_011657992.1	auxin response factor 7-like	−1.30	2114
csa-miR160a	XM_004150892.2	auxin response factor 7-like	−1.14	1568
csa-miR160a	XM_004143204.2	auxin response factor 7-like	−0.87	2118
csa-miR160a	NM_001288596.1	auxin response factor 7	−0.22	1346
csa-miR160a	NM_001281787.1	auxin response factor 7-like	−1.30	1334
cme-miR164a	XM_011661856.1	NAC domain-containing protein 89-like	4.02	828
cme-miR164a	XM_011656041.1	exopolygalacturonase-like	-	N
cme-miR164a	XM_011653931.1	NAC domain-containing protein 89-like	−2.10	861
cme-miR164a	XM_011653227.1	NAC domain-containing protein 89-like	−0.85	730
cme-miR164a	XM_011652994.1	NAC domain-containing protein 89-like	-	803
cme-miR164a	XM_011651827.1	disease resistance protein RPM1	-	N
cme-miR164a	XM_004150728.2	NAC domain-containing protein 89-like	−0.85	739
cme-miR164a	XM_004146051.2	NAC domain-containing protein 89-like	-	960
cme-miR164a	XM_004141527.2	NAC domain-containing protein 89-like	-	N
cme-miR164a	XM_004137540.2	NAC domain-containing protein 89-like	−0.90	970
cme-miR164a	XM_004136650.2	NAC domain-containing protein 89-like	−2.10	864
cme-miR164a	XM_004136327.2	1-phosphatidylinositol-3-phosphate 5-kinase FAB1B-like	-	322
csa-MIR166d	XM_011652521.1	putative ABC transporter C family member 15	-	N
cme-miR399c	XM_011659907.1	probable ubiquitin-conjugating enzyme E2 24	−0.82	473
cme-miR399c	XM_004134656.2	probable ubiquitin-conjugating enzyme E2 24	−0.82	714
csa-mir013	XM_011661833.1	calcium-dependent protein kinase 8	-	N
csa-mir013	XM_011661179.1	myosin class 11-1 (ISS)	-	N
csa-mir013	XM_011659724.1	cell division control-like protein	-	N
csa-mir013	XM_011659130.1	probable L-ascorbate peroxidase 6	−0.47	1272
csa-mir013	XM_011658857.1	chitin elicitor receptor kinase 1-like	-	N
csa-mir013	XM_011656847.1	glutamate decarboxylase 1	-	268
csa-mir013	XM_011652440.1	mitogen-activated protein kinase homolog D5-like	-	N
csa-mir013	XM_011651425.1	NADP-dependent malic enzyme	-	N
csa-mir013	XM_011650949.1	THO complex subunit 3	-	N
csa-mir013	XM_004150014.2	THO complex subunit 3	-	N
csa-mir013	XM_004149751.2	probable L-ascorbate peroxidase 6	−0.47	1273
csa-mir013	XM_004148951.2	omega-hydroxypalmitate O-feruloyl transferase-like	-	N
csa-mir013	XM_004148279.2	homogentisate phytyltransferase 1	-	N
csa-mir013	XM_004144564.2	exopolygalacturonase clone GBGA483	-	N
csa-mir013	XM_004143155.2	chitin elicitor receptor kinase 1-like	-	N
csa-mir013	XM_004137239.2	putative uncharacterized protein At4g01020	-	N
csa-mir013	XM_004135249.2	signal recognition particle subunit SRP72-like	-	1800
csa-mir013	XM_004133898.2	ent-kaurenoic acid oxidase 1	-	N

Note: log_2_ (Fold Change) indicates log_2_ (Fold_change) of miRNA expression between EXP_5d vs. uE_0d or EXP_5d vs. uE_5d sRNA libraries; “-” indicates that the transcript is only identified in one sRNA library among uE_0d, uE_5d and EXP_5d; “N” indicates no degradome cleavage site is detected.

## Supplementary Materials

The following are available online at https://www.mdpi.com/2218-273X/9/9/483/s1. Table S1: The sequences of miRNAs identified in cucumber fruit, Table S2: Differentially expressed miRNAs between EXP_5d vs. uE_0d, EXP_5d vs. uE_5d and uE_5d vs. uE_0d, respectively, Table S3: Overview of sequencing data generated from degradome sequencing, Table S4: Target genes of identified differentially expressed miRNAs.
